# Previous uterine artery embolization increases the rate of repeat embolization in a subsequent pregnancy

**DOI:** 10.1371/journal.pone.0185467

**Published:** 2017-09-26

**Authors:** Geum Joon Cho, Jae-Yoon Shim, Yung-Taek Ouh, Log Young Kim, Tae Seon Lee, Ki Hoon Ahn, Soon-Cheol Hong, Min-Jeong Oh, Hai-Joong Kim, Pil Ryang Lee

**Affiliations:** 1 Department of Obstetrics and Gynecology, Korea University Guro Hospital, Korea University College of Medicine, Seoul, Korea; 2 Department of Obstetrics and Gynecology, Asan Medical Center, University of Ulsan College of Medicine, Seoul, Korea; 3 The Health Insurance Review & Assessment Service of Korea, Seoul, Korea; Academic Medical Centre Amsterdam, NETHERLANDS

## Abstract

This study aimed to determine the rate of repeat uterine artery embolization (UAE) in women with a previous UAE. Study data were collected from the Korea National Health Insurance Claims Database of the Health Insurance Review and Assessment Service for 2009–2013. We enrolled women who had a first delivery in 2009 and a second delivery between 2010 and 2013. Among 226,408 women who had a first delivery in 2009, 296 underwent UAE. A total of 127,506 women had a second delivery between 2010 and 2013. Of 296 women who underwent UAE after the first delivery, 94 had a second delivery between 2010 and 2013. Women with a previous UAE had a higher rate of UAE at the second delivery than women without a previous UAE. Multivariate adjusted analysis showed that a UAE at the first delivery increased the rate of UAE at the second delivery (odds ratio 25.56, 95% confidence interval 9.86–66.23). Women with a previous UAE should be appropriately counseled and monitored for the need for a repeat UAE.

## Introduction

Postpartum hemorrhage (PPH) is dangerous, and is a leading cause of maternal morbidity and mortality [1–3]. In cases of uncontrolled PPH despite medical treatment, conservative surgical management can be tried. Among several treatment options for uncontrolled PPH, uterine artery embolization (UAE) is a safe and minimally invasive procedure with a greater than 90% success rate for adequate hemostasis [4–6]. A major advantage of UAE in the treatment of PPH is its potential to preserve fertility by avoiding hysterectomy [7]. The need for UAE is associated with many risk factors, including older maternal age, multiple pregnancies, cesarean section, labor induction, instrumental delivery, and placenta previa [8].

The rate of UAE for PPH has increased [8], and subsequent pregnancy outcomes are of concern in treated women. Several studies have evaluated the long-term effect of UAE on fertility and future pregnancy outcomes, and found that these women can conceive and have normal pregnancies [9–12]. While subsequent pregnancies are clearly possible, there are reports of recurrent severe PPH at subsequent deliveries (31.6–100%) [10, 12]. However, it is not known whether women with a previous UAE have a higher rate of repeat UAE in a subsequent pregnancy. As UAE is not always appropriate, successful, or available, this information is essential for counseling women with a previous UAE about subsequent pregnancies, and for the obstetrician to plan appropriate management. Our study aimed to determine the rate of repeat UAE in women with a previous UAE.

## Methods

The KNHI program enrolls 97% of the Korean population. The current health insurance policy requires healthcare providers to permit HIRA to audit medical insurance claims. The remaining 3% of the population is under the Medical Aid Program. Therefore, the HIRA database consists of medical insurance claims data for nearly 50 million Koreans, but excludes uninsured services such as cosmetic surgery. The database contains information on the incidence of nearly all diseases occurring in Korea. HIRA provides data for research to researchers. The characteristics of this research data in other study are well described [13]. In brief, HIRA research data consists of 6 files: 1) the general information file; 2) the healthcare services file, including inpatient prescriptions; 3) the diagnoses file; 4) the outpatient prescriptions file; 5) the drug master file; and 6) the provider information file. The healthcare services file houses specific and detailed information for healthcare services provided to beneficiaries such as procedures, diagnostic tests, treatments, and inpatient prescriptions. The diagnosis file includes records of all diagnoses that a beneficiary has received. This is also the case for pregnancy, so all the diagnoses and procedures related to pregnancy can be confirmed. Especially, the billing statement codes are different depending on whether it is primiparous or multiparous, singleton or multiple pregnancy, vaginal delivery or cesarean section. Thus, these variables can be confirmed accurately. The Act on the Protection of Personal Information Maintained by Public Agencies permits use of the claims data if individual identifiers are concealed. We received the database including an unidentifiable code for each individual, but age, diagnosis, and a list of provided procedures were available.

We identified all women who delivered and women who underwent peripartum hysterectomy (PH) or UAE during the study period by using the International Classification of Diseases, tenth Revision (ICD-10) diagnosis and procedure codes. PH refers to either a total or subtotal abdominal hysterectomy during vaginal or cesarean delivery. UAE refers to performance of UAE during hospitalization for delivery. We excluded patients with a concomitant diagnosis of malignancy from the analysis. A first pregnancy was matched with a second pregnancy during the study period. We included women with a first delivery in 2009 and a subsequent delivery between 2010 and 2013.

We compared continuous variables between groups using Student’s t-test, and categorical variables using the chi-square test. In order to evaluate risk, a model of multivariate logistic regression analysis was performed with UAE in the second pregnancy as the final outcome for the entire study population. Statistical significance was defined as a *P* value < 0.05. Statistical analyses were performed using SPSS software, version 12.0 (SPSS Inc., Chicago, IL, USA).

## Results

Among 935,209 women who had a first delivery between 2009 and 2013, 1,222 (0.13%) underwent UAE and 690 (0.07%) underwent PH (Table 1).

**Table 1:** Basic characteristics of women who had a first delivery in 2009 (n = 935,209).

Variables	
Age (years)	29.40 ± 3.99
Older maternal age (≥ 35 years) (%)	85,134 (9.10%)
Multiple pregnancies (%)	19,170 (2.05%)
Cesarean section (%)	341,105 (36.47%)
Preeclampsia (%)	24,159 (2.58%)
Induction of labor (%)	224,408 (24.00%)
Placenta previa (%)	8,607 (0.92%)
Placental abruption (%)	4,351 (0.47%)
PPH (%)	44,747 (7.14%)
UAE (%)	1,222 (0.13%)
PH (%)	690 (0.07%)

Results are presented as mean ± standard deviation (SD) or as n (%). PPH, postpartum hemorrhage; UAE, uterine arterial embolization; PH, peripartum hysterectomy

Of these 935,209 women, 317,670 had a second delivery between 2009 and 2013. Of the 1,222 who underwent UAE at the first delivery, 217 had a second delivery between 2009 and 2013 showing a significantly lower percentage of women who underwent UAE in their initial pregnancy had a subsequent pregnancy, compared with women without UAE (17.8% vs 34.0%, P < .01).

Table 2 shows demographic information for the study population in a subsequent pregnancy, according to a history of UAE in the first pregnancy. Compared to women without UAE at the first delivery, women with previous UAE had higher rates of older maternal age, cesarean section, preeclampsia, placenta previa, placental abruption, and PPH in a subsequent pregnancy and had a longer interval between first and second pregnancies. Of those with a history of UAE at the first delivery, 13 underwent UAE and 11 underwent PH, showing a higher rate than that for women without previous UAE (Table 2).

**Table 2:** Basic characteristics of the study population in a second pregnancy, according to a history of UAE at the first delivery (n = 317,670).

	History of UAE in first pregnancy		*P*-value
	No (317,453)	Yes (217)	
Age (years)	31.07 ± 3.53	32.51 ± 3.12	< .01
Interval between first and second pregnancies (years)	2.23 ± 0.74	2.34 ± 0.76	.02
Older maternal age (≥ 35 years) (%)	45 901 (14.46)	54 (24.88)	< .01
Multiple pregnancies (%)	2750 (0.87)	4 (1.84)	.12
Cesarean section (%)	108 419 (34.15)	115 (53.00)	< .01
Preeclampsia (%)	3341 (1.05)	10 (4.61)	< .01
Induction of labor (%)	71 664 (22.57)	48 (22.12)	.87
Placenta previa (%)	2070 (0.65)	20 (9.21)	< .01
Placental abruption (%)	768 (0.24)	2 (0.92)	.04
PPH (%)	22 042 (6.94)	55 (25.35)	< .01
UAE (%)	328 (0.10)	13 (5.99)	< .01
PH (%)	204 (0.06)	11 (5.07)	< .01

Results are presented as mean ± SD or as n (%). UAE, uterine arterial embolization; PPH, postpartum hemorrhage; PH, peripartum hysterectomy

Multivariate adjusted odds ratio (ORs) for UAE are shown in Table 3. Older maternal age, interval between first and second pregnancies, multiple pregnancies, induction of labor, placenta previa, placental abruption, PPH, and UAE in the first pregnancy were associated with an increased risk of UAE. The risk was highest in women with PPH (OR 24.72, 95% confidence interval [CI] 19.66–31.08).

**Table 3:** Odds ratios (OR) and 95% confidence interval (CI) for the risk of UAE in the second pregnancy.

	Unadjusted OR(95% CI)	Adjusted OR^a^ (95% CI)
Older maternal age	1.73 (1.34, 2.23)	1.43 (1.09, 1.87)
Interval between first and second pregnancies	1.26 (1.10, 1.44)	1.18 (1.03, 1.36)
Multiple pregnancies	5.29 (3.15, 8.88)	4.99 (2.89, 8.61)
Cesarean delivery	1.38 (1.11, 1.71)	0.88 (0.66, 1.17)
Preeclampsia	3.13 (1.72, 5.72)	1.80 (0.93, 3.48)
Induction of labor	1.19 (0.94, 1.52)	1.36 (1.03, 1.80)
Placenta previa	29.79 (22.25, 39.89)	20.25 (14.17, 28.94)
Placental abruption	15.23 (8.53, 27.22)	7.08 (3.63, 13.81)
PPH	27.63 (22.03, 34.65)	24.72 (19.66, 31.08)
UAE in first pregnancy	61.61 (34.81 109.06)	12.46 (5.96, 26.03)

OR, odds ratio; CI, confidence interval; UAE, uterine arterial embolization, PPH, postpartum hemorrhage

OR^a^s were adjusted for all variables in the table.

## Discussion

We believe this study is the first to report the rate of repeat UAE in a subsequent pregnancy in women who underwent a previous UAE. Women with UAE in the first pregnancy had an increased rate of repeat UAE in a subsequent pregnancy, compared with those without previous UAE. Although the reasons for this association are not clear, several explanations are possible. First, PPH in a previous pregnancy, a major indication for UAE, is often associated with recurrent PPH in a subsequent pregnancy [14–17]; the association remains significant regardless of the delivery mode in a previous pregnancy [14]. Salomon et al. reported that 4 patients with a previous UAE delivered healthy babies, but PPH recurred in all, and led to hysterectomy in 2 patients. They suggested that uterine damage due to UAE, through an unknown mechanism, might lead to abnormal placentation, causing PPH [10]. Sentilhes et al. also reported recurrence of PPH in 6 of 19 pregnancies in women with a prior history, and suggested that these women should be considered to have an increased risk of PPH in future deliveries [12]. Therefore, PPH in women with a previous UAE is more likely to recur in a subsequent pregnancy and is also more likely to require UAE, an invasive procedure. Second, women with a history of PPH, in particular severe cases requiring invasive procedures including UAE, may have significant anxiety throughout pregnancy [18]. As obstetricians may also have anxiety, they monitor these women for recurrence of PPH in a subsequent pregnancy. Therefore, it is possible that the diagnosis is made more frequently during the second pregnancy in women with a history of PPH, and it is thus likely that UAE will be used more often in a subsequent pregnancy, with a low threshold for a prompt aggressive response. Further studies are needed to ascertain the reasons for an increased rate of repeat UAE.

Our results have critical implications in guiding clinical treatment. Women with a previous UAE were at elevated risk of invasive procedures, including UAE (13/217) and PH (11/217) in subsequent pregnancies. A radiologist trained to perform UAE and a shielded suite may be unavailable in a community hospital [7]. Thus, women with a previous UAE should be counseled about the possibility of a repeat UAE, and may be referred appropriately to a high-risk medical center for delivery, a known means of improving outcomes in certain obstetric conditions [19–21].

In this study, a significantly lower percentage of women who underwent UAE in their initial pregnancy had a subsequent pregnancy, compared with women without UAE. This may be because women with a previous severe PPH tended to avoid another pregnancy because of fear of recurrence. Despite preservation of the uterus, severe PPH is reported to have a long-term psychological effect on patients; the psychological effect of severe PPH may be greater after UAE than that after other surgical procedures [18]. However, it has been reported that PPH in the first pregnancy was not associated with either a difference in the rate of second pregnancies or the time interval between the first and second pregnancy [22]. Women with PPH associated with cesarean delivery in a first pregnancy were less likely to have a second pregnancy, suggesting that factors other than delivery method may be more relevant than the history of PPH itself [22]. However, we were unable to evaluate the conception rate in second pregnancies due to limited of data. Further studies are required to confirm the long-term effect of UAE on conception.

In this study, cesarean section was associated with UAE in subsequent pregnancy but lost statistical significance after adjustment for other variables (Table 1) in contrast with results from our previous study [8]. The reason is unclear, but there are some plausible explanations. First, several factors, which are known to be associated with cesarean section and risk factors for UAE, are controlled in this study. Thus, cesarean section lost statistical significance after these factors. Another reason for this association may be due to the study design. In this study, we only enrolled pregnant women with the second delivery. Thus, in some obstetric situations with the potential for massive hemorrhage, planned PH is the preferred delivery strategy rather than UAE. Therefore, cesarean section may be not associated with UAE in the second delivery.

There are some limitations in this study. First, the incidence of UAE in this study was derived from the KNHI Claims Database, which was designed to collect data on the cost of claims, rather than to aid research. Thus, a main limitation is the validity of the data. However, the accuracy of diagnostic codes tended to be higher for claims for more severe conditions [23, 24]. This would be the diagnoses that cause UAE. Moreover, Claims are electronically submitted by providers to HIRA which reviews claims and makes reimbursement decisions. In addition, claimed items and cost are provided to the beneficiaries suggesting that the chances of missing claims for procedure are highly unlikely [13]. It has been also reported the claims database has a higher reliability than other databases for estimating healthcare utilization [25], such as UAE procedures. Another limitation is that we did not have information on the indications for UAE and other factors related to PPH, because this is not available in the database. Thus, UAE may have been performed for other causes such as myomas rather than for PPH. However, in this study, UAE was limited to performance of UAE during hospitalization for delivery. Although we did not look at all of accompanying diagnoses except the diagnosis related UAE performed during deliveries, it is unlikely that UAE was performed during hospitalization for delivery in the treatment of other causes such as myomas. Type of institution may be an important factor for determining UAE, because, in institution where the resources are available, it is likely that UAE will be used with a low threshold for a prompt aggressive response. Similar to the type of institution, the place of birth will be also important. Women born in place with radiology facility will likely have a UAE rather than PH. Conversely, if women are born in a place where there is no radiology facilities, women will be receiving a PH or being transferred to another institution. Unfortunately, data of type of institution and place of birth are unavailable. However, compared to women without UAE at the first delivery, women with previous UAE had higher rates of PH as well as UAE suggesting if UAE could have been properly performed to these high risk women, it may have reduced unnecessary PH. The aim of this study was to determine whether women with a previous UAE is at risk for invasive procedure in subsequent pregnancy. Thus, the influence of type of institution is considered to be minimal.

Nevertheless, the strength of the present study lies in the evaluation of data from a population-based registry containing information regarding all births and procedures related to pregnancy in Korea, during the time period considered.

## Conclusions

We observed an increased rate of repeat UAE in women with a previous UAE. Women with a previous UAE should be appropriately counseled and monitored the need for a repeat UAE.

## Abbreviations

PPH: postpartum hemorrhage
UAE: uterine artery embolization
PH: peripartum hysterectomy

## References

[1] Khan KS, Wojdyla D, Say L, Gülmezoglu AM, Van Look PF. WHO analysis of causes of maternal death: a systematic review. Lancet. 2006;367(9516):1066–74. Epub 2006/04/04. doi: 10.1016/S0140-6736(06)68397-9 . 16581405 10.1016/S0140-6736(06)68397-9

[2] Berg CJ, Callaghan WM, Syverson C, Henderson Z. Pregnancy-related mortality in the United States, 1998 to 2005. Obstet Gynecol. 2010;116(6):1302–9. Epub 2010/11/26. doi: 10.1097/AOG.0b013e3181fdfb11 . 21099595 10.1097/AOG.0b013e3181fdfb11

[3] King JF, Slaytor EK, Sullivan EA. Maternal deaths in Australia, 1997–1999. Med J Aust. 2004;181(8):413–4. Epub 2004/10/19. . 15487953 10.5694/j.1326-5377.2004.tb06361.x

[4] Sidhu HK, Prasad G, Jain V, Kalra J, Gupta V, Khandelwal N. Pelvic artery embolization in the management of obstetric hemorrhage. Acta Obstet Gynecol Scand. 2010;89(8):1096–9. Epub 2010/04/20. doi: 10.3109/00016349.2010.481015 . 20397757 10.3109/00016349.2010.481015

[5] Pelage JP, Le Dref O, Mateo J, Soyer P, Jacob D, Kardache M, et al Life-threatening primary postpartum hemorrhage: treatment with emergency selective arterial embolization. Radiology. 1998;208(2):359–62. Epub 1998/07/29. doi: 10.1148/radiology.208.2.9680559 . 9680559 10.1148/radiology.208.2.9680559

[6] Shim JY, Yoon HK, Won HS, Kim SK, Lee PR, Kim A. Angiographic embolization for obstetrical hemorrhage: effectiveness and follow-up outcome of fertility. Acta Obstet Gynecol Scand. 2006;85(7):815–20. Epub 2006/07/04. doi: 10.1080/00016340500438652 . 16817079 10.1080/00016340500438652

[7] Hunter LA. Exploring the role of uterine artery embolization in the management of postpartum hemorrhage. J Perinat Neonatal Nurs. 2010;24(3):207–14. Epub 2010/08/11. doi: 10.1097/JPN.0b013e3181e8c994 . 20697237 10.1097/JPN.0b013e3181e8c994

[8] Cho GJ, Kim LY, Hong HR, Lee CE, Hong SC, Oh MJ, et al Trends in the rates of peripartum hysterectomy and uterine artery embolization. PLoS One. 2013;8(4):e60512 doi: 10.1371/journal.pone.0060512 ; PubMed Central PMCID: PMC3615013. 23565254 10.1371/journal.pone.0060512 PMC3615013

[9] Descargues G, Mauger Tinlot F, Douvrin F, Clavier E, Lemoine JP, Marpeau L. Menses, fertility and pregnancy after arterial embolization for the control of postpartum haemorrhage. Hum Reprod. 2004;19(2):339–43. Epub 2004/01/30. . 14747177 10.1093/humrep/deh082

[10] Salomon LJ, deTayrac R, Castaigne-Meary V, Audibert F, Musset D, Ciorascu R, et al Fertility and pregnancy outcome following pelvic arterial embolization for severe post-partum haemorrhage. A cohort study. Hum Reprod. 2003;18(4):849–52. Epub 2003/03/28. . 12660283 10.1093/humrep/deg168

[11] Stancato-Pasik A, Mitty HA, Richard HM 3rd, Eshkar N. Obstetric embolotherapy: effect on menses and pregnancy. Radiology. 1997;204(3):791–3. Epub 1997/09/01. doi: 10.1148/radiology.204.3.9280261 . 9280261 10.1148/radiology.204.3.9280261

[12] Sentilhes L, Gromez A, Clavier E, Resch B, Verspyck E, Marpeau L. Fertility and pregnancy following pelvic arterial embolisation for postpartum haemorrhage. BJOG. 2010;117(1):84–93. Epub 2009/10/17. doi: 10.1111/j.1471-0528.2009.02381.x . 19832826 10.1111/j.1471-0528.2009.02381.x

[13] Kim JA, Yoon S, Kim LY, Kim DS. Towards Actualizing the Value Potential of Korea Health Insurance Review and Assessment (HIRA) Data as a Resource for Health Research: Strengths, Limitations, Applications, and Strategies for Optimal Use of HIRA Data. J Korean Med Sci. 2017 5;32(5):718–728. doi: 10.3346/jkms.2017.32.5.718 28378543 10.3346/jkms.2017.32.5.718 PMC5383602

[14] Buzaglo N, Harlev A, Sergienko R, Sheiner E. Risk factors for early postpartum hemorrhage (PPH) in the first vaginal delivery, and obstetrical outcomes in subsequent pregnancy. J Matern Fetal Neonatal Med 2015;28(8):932–7. doi: 10.3109/14767058.2014.937698 25023434 10.3109/14767058.2014.937698

[15] Hall MH, Halliwell R, Carr-Hill R. Concomitant and repeated happenings of complications of the third stage of labour. Br J Obstet Gynaecol. 1985;92(7):732–8. Epub 1985/07/01. . 3874647 10.1111/j.1471-0528.1985.tb01456.x

[16] Ford JB, Roberts CL, Bell JC, Algert CS, Morris JM. Postpartum haemorrhage occurrence and recurrence: a population-based study. Med J Aust. 2007;187(7):391–3. Epub 2007/10/03. . 17908001 10.5694/j.1326-5377.2007.tb01308.x

[17] Oberg AS, Hernandez-Diaz S, Palmsten K, Almqvist C, Bateman BT. Patterns of recurrence of postpartum hemorrhage in a large population-based cohort. Am J Obstet Gynecol. 2014;210(3):229.e1–8. Epub 2013/12/20. doi: 10.1016/j.ajog.2013.10.872 ; PubMed Central PMCID: PMCPmc3943527. 24351791 10.1016/j.ajog.2013.10.872 PMC3943527

[18] Sentilhes L, Gromez A, Clavier E, Resch B, Descamps P, Marpeau L. Long-term psychological impact of severe postpartum hemorrhage. Acta Obstet Gynecol Scand. 2011;90(6):615–20. Epub 2011/03/05. doi: 10.1111/j.1600-0412.2011.01119.x . 21370999 10.1111/j.1600-0412.2011.01119.x

[19] Eller AG, Bennett MA, Sharshiner M, Masheter C, Soisson AP, Dodson M, et al Maternal morbidity in cases of placenta accreta managed by a multidisciplinary care team compared with standard obstetric care. Obstet Gynecol. 2011;117(2 Pt 1):331–7. Epub 2011/02/12. . 21309195 10.1097/AOG.0b013e3182051db2

[20] Wright JD, Herzog TJ, Shah M, Bonanno C, Lewin SN, Cleary K, et al Regionalization of care for obstetric hemorrhage and its effect on maternal mortality. Obstet Gynecol. 2010;115(6):1194–200. Epub 2010/05/27. doi: 10.1097/AOG.0b013e3181df94e8 . 20502290 10.1097/AOG.0b013e3181df94e8

[21] Shim JY, Hong SY, Won HS, Lee PR, Kim A. Conservative multidisciplinary management of placenta percreta following in vitro fertilization. Obstet Gynecol Sci. 2013;56(3):194–7. Epub 2013/12/12. doi: 10.5468/ogs.2013.56.3.194 ; PubMed Central PMCID: PMC3784122. 24328001 10.5468/ogs.2013.56.3.194 PMC3784122

[22] Fullerton G, Danielian PJ, Bhattacharya S. Outcomes of pregnancy following postpartum haemorrhage. BJOG. 2013;120(5):621–7. Epub 2013/01/24. doi: 10.1111/1471-0528.12120 . 23339709 10.1111/1471-0528.12120

[23] Kim JY. Strategies to enhance the use of National Health Insurance claims database in generating health statistics Seoul: Health Insurance Review and Assessment Services; 2005.

[24] Park BJ, Sung JH, Park KD, Seo SW, Kim SW. Strategies to improve the validity of diagnostic codes of National Health Insurance claims data Seoul: Health Insurance Review and Assessment Services; 2002 118–9 p.

[25] Kang HY, Lim SJ, Suh HS, Liew D. Estimating the lifetime economic burden of stroke according to the age of onset in South Korea: a cost of illness study. BMC Public Health. 2011;11:646 Epub 2011/08/16. doi: 10.1186/1471-2458-11-646 ; PubMed Central PMCID: PMCPmc3171726. 21838919 10.1186/1471-2458-11-646 PMC3171726

